# Scripting Analyses of Genomes in Ensembl Plants

**DOI:** 10.1007/978-1-0716-2067-0_2

**Published:** 2022-01-01

**Authors:** Bruno Contreras-Moreira, Guy Naamati, Marc Rosello, James E. Allen, Sarah E. Hunt, Matthieu Muffato, Astrid Gall, Paul Flicek

**Keywords:** Database, Genomics, Comparative genomics, Genetic variation, Crops, Model plants, Polyploids, Scripting, API

## Abstract

Ensembl Plants (http://plants.ensembl.org) offers genome-scale information for plants, with four releases per year. As of release 47 (April 2020) it features 79 species and includes genome sequence, gene models, and functional annotation. Comparative analyses help reconstruct the evolutionary history of gene families, genomes, and components of polyploid genomes. Some species have gene expression baseline reports or variation across genotypes. While the data can be accessed through the Ensembl genome browser, here we review specifically how our plant genomes can be interrogated programmatically and the data downloaded in bulk. These access routes are generally consistent across Ensembl for other non-plant species, including plant pathogens, pests, and pollinators.

## 1 Introduction

Plants play a central role in the ecology and economy of our planet and are essential to our food security. As the world population increased by 145% in the last 60 years, the yields of cereals increased even more, while not needing much more land [1]. This has been possible as a result of improved agricultural practices and crops. Currently, breeding programs take advantage of inexpensive genomic and phenotypic data. The next steps towards what is being called Breeding 4.0 [2] include adapting crops to changing environments and broadening the diversity pool to compensate for the losses occurred during domestication. For this reason wild relatives of crops are being sequenced increasingly and added to pre-breeding programs [3]. In addition, natural plant populations and model plants are being studied to understand their ecology and the genetic basis of their adaptation mechanisms, which can then be applied to in crops. In this context, genomics is a foundation of plant sciences, as standard approaches such as marker-assisted breeding, QTL analysis, and genome-wide association studies, as well as genomic selection, induced variation experiments, and genome editing, all depend on genomic technologies and databases. These tools are accelerating breeding and helping to untangle complex polyploid genomes, such as that of bread wheat [4].

Ensembl Plants (http://plants.ensembl.org) is the Ensembl portal for plants and red algae [5] and provides a consistent set of interfaces to genomic data, including reference genome sequences, gene and transcript models, genetic variation, gene expression, markers, and comparative genomics. There are up to four releases per year. At the time of writing, the latest release of Ensembl Plants is version 47 (April 2020), which corresponds to Ensembl version 100. This release comprises 79 genomes, containing several cultivars and ecotypes for some species. Ensembl Plants is developed with our long-term partners Gramene [6] and with individual groups that publish plant genomes around the world. This chapter documents how the data at Ensembl Plants can be downloaded in bulk and interrogated programmatically using a variety of approaches. It provides a series of recipes, available as source code at https://github.com/Ensembl/plant-scripts, that can be modified to carry out more complex analyses of plant genomes.

## 2 Materials

### 2.1 Database Structure and Data Access

Ensembl Plants is implemented primarily as a collection of MySQL relational databases. The overall data structure is modular, with different data (e.g., core annotation, comparative genomics, functional genomics, variation data) modeled by distinct schemas. The core schema is modeled on the central dogma of molecular biology, linking genome sequence to genes, transcripts, and their translations, each of which can be decorated with functional annotation (*see* Note 1). Much annotation takes the form of cross-references, which are web links to entries in other resources, such as InterPro [7] or Gene Ontology [8], that either represent the primary source of the biological entity or provide additional information. Cross-references describe functional entities such as domains, reactions, and processes. Some also serve as controlled vocabularies for functional annotation.

The databases can be downloaded for local installation or alternatively accessed via a public MySQL server. Local MySQL databases are an efficient alternative to the public MySQL server, particularly if heavy use is anticipated (*see* Note 2). Programmatic access is supported by two APIs, which allow data discovery and access through an abstraction layer that hides the detailed structure of the underlying data store. One is a Perl API, while the other uses a language-agnostic REST interface [9]. The REST service allows up to 15 requests per second.

In addition to the primary databases, Ensembl Plants also provides access to denormalized data warehouses, constructed using the BioMart tool kit [10]. These are specialized databases that support efficient gene- and variant-centric queries. Finally, a variety of data selections are exported from the databases in common file formats and made available for download via an FTP site.

These resources are summarized in Table 1. Recipes to query each of them are listed in Table 7.

### 2.2 Overview of Data Content

#### 2.2.1 Genomes and Core Data

Genome assemblies are typically imported from the European Nucleotide Archive (ENA) [11], which is part of the International Nucleotide Sequence Database Collaboration (http://www.insdc.org, INSDC). Gene model annotations are imported from the ENA [11], Phytozome [12], or provided by community members (see Note 3). For instance, the rice annotation was imported from RAP-DB [13]. After import, various computational analyses are performed for each genome. A summary of these is given in Table 2. In addition, specific datasets are imported and analyzed according to the requirements of individual communities. These datasets typically fall into two classes, markers, and variants across genotype panels.

The genomes currently included in Ensembl Plants are listed in Table 3. A summary of UniProt coverage of proteins encoded by genes within these genomes is given in Table 4 [17]. In all cases, genomes are identified by their Ensembl production name, which is usually binomial but can also include a strain name to distinguish particular cultivars or ecotypes, such as *malus_domestica_golden*. Details of other datasets incorporated can be found through the homepage for each species (*see* Note 3).

#### 2.2.2 Variation Data

The variation schema can store genetic variants observed in populations or germplasm collections, alleles, and frequencies, alongside sample genotype data. Supported variant types include single nucleotide polymorphisms, indels, and structural variants. The functional consequence of variants on genes is predicted with the Ensembl Variant Effect Predictor (VEP) [14]. Linkage disequilibrium data and statistical associations with phenotypes are available for selected species. The variation datasets of release 47 of Ensembl Plants are described in Table 5. The Ensembl VEP is also a command line tool that can be used to efficiently annotate variants and we provide recipes for it as well (*see* Table 7).

#### 2.2.3 Comparative Genomics Data

**Fig. 1:**
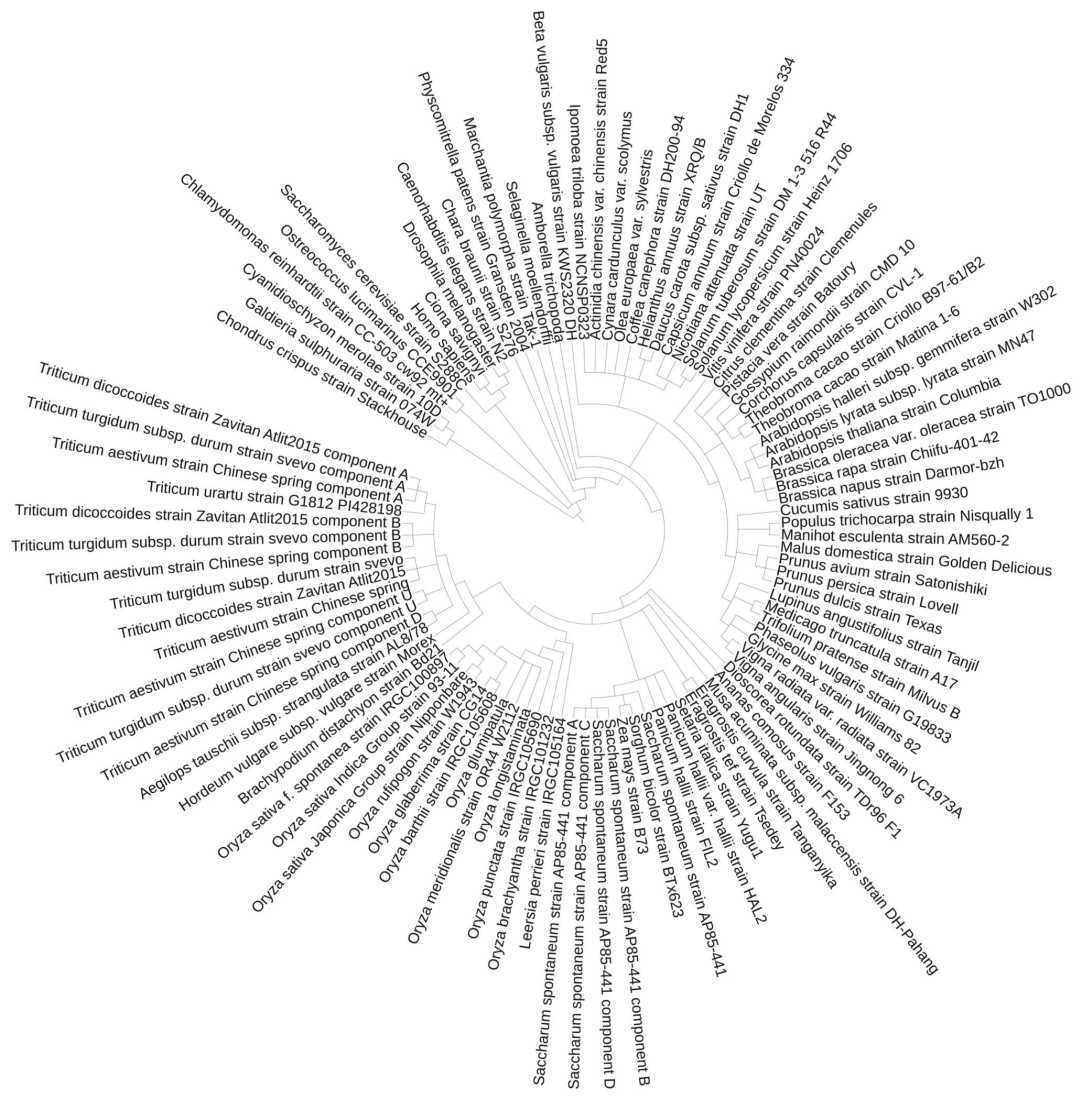
Species cladogram of release 47 (April 2020) of Ensembl Plants. Genomes of polyploid species are decomposed into genomic components. This topology is used in the comparative genomic analyses to derive orthologous and paralogous genes. This tree was produced with the Newick file obtained with recipe F12 and visualized with iToL [41]

The Ensembl Gene Tree pipeline is used to calculate evolutionary relationships among members of protein families (Table 2). For each gene, the translation of the canonical transcript is selected (*see* Note 4). Briefly, this pipeline first finds clusters of similar proteins and then, for each cluster, attempts to reconcile the relationship between the sequences with the known species cladogram (Fig. 1), derived from the NCBI Taxonomy database [42]. The analysis also contains a few non-plant outgroups. The TreeBeST software (https://github.com/Ensembl/treebest) is used to construct a consensus tree, which allows the identification of orthologues and paralogues. As polyploid genomes are split into components, homoeologous genes are effectively defined as orthologues among subgenomes. A number of plant genomes are also included in a pan-taxonomic gene tree, containing a representative selection of sequenced genomes from all domains of life. Recipe R2 can be used to check which comparative analyses have been run for a particular species. This information is also displayed in the table at http://plants.ensembl.org/species.html.

Other comparative analyses available in Ensembl Plants are pairwise whole-genome alignments and synteny (*see* Tables 2 and 6).

#### 2.2.4 Baseline Expression Data

Baseline gene expression reports are available as “Gene expression” on the website for selected species. An example for barley is shown at http://plants.ensembl.org/Hordeum_vulgare/Gene/ExpressionAtlas?g=HORVU5Hr1G095630;r=chr5H:599085656-599133086. The underlying curated expression data, produced by Expression Atlas [44], can be browsed and downloaded via the expression widget.

#### 2.2.5 RNA-seq Tracks

RNA-seq datasets from the public INSDC archives are mapped to genome assemblies in Ensembl Plants in every release. They are handled as ENA studies and for each of them CRAM files are created with the RNA-Seq-er pipeline (https://www.ebi.ac.uk/fg/rnaseq/api) [45] and published at ftp://ftp.ensemblgenomes.org/pub/misc_data/Track_Hubs. Each study contains a separate folder for each assembly that was used for mapping. These tracks can be interactively displayed in the browser, but can be of interest for high-throughput studies as well. For instance, study SRP133995 was mapped to tomato assembly SL3.0 and the tracksDb.txt file therein indicates the full path to the relevant CRAM file next to its metadata. CRAM files for a selected assembly can be discovered with recipe C1; note that the assembly name corresponds to column “assembly_default” in recipe R2. As of May 2020 there were 89,355 CRAM files available.

## 3 Methods

This section describes some of the recipes listed in Table 7 in detail so that the reader can execute or modify any of them. Software dependencies required by these recipes are listed in https://github.com/Ensembl/plant-scripts/blob/master/README.md.

The different approaches are complementary. While the native Perl API is the most powerful and used extensively by Ensembl developers, it also requires some Perl knowledge and the installation of several repositories. Similarly, the Biomart and MySQL examples require knowledge of R and SQL, respectively. However, the REST endpoints can be interrogated with any programming language; however, only a defined set of queries are currently supported. The FTP recipes allow efficient bulk downloads, but with no customization. The source code for all recipes can be found at https://github.com/Ensembl/plant-scripts.

### 3.1 Clone the GitHub Repository and Install Dependencies

The following steps explain how to obtain a local copy of the recipes and how to test them on Linux/MacOS operating systems (OS).

1. Open a terminal and check whether *git* is installed by typing: git --version.
2. If required install *git* if using the appropriate software manager for your OS.
3. Clone the repository: git clone https://github.com/Ensembl/plant-scripts.git.
4. Navigate to the scripts directory: cd plant-scripts.
5. Optionally test the scripts: perl demo_test.t.

### 3.2 Perl API Recipes

The Ensembl Perl API enables access to all types of data from Ensembl Plants (genes, variation, comparative genomics, regulation, etc.) and it is documented extensively (*see* Note 5). It allows complex queries to be executed without the construction of any explicit SQL queries. The repository contains eight Perl API recipes, of which three are described here (A1, A4, and A8).

#### 3.2.1 Get a BED File with Repeats on Chromosome 4

1. Load the Registry object with details of genomes available from the public Ensembl Genomes servers (recipe A1):
Use Bio::EnsEMBL::Registry;
                                          Bio::EnsEMBL::Registry->load_registry_from_db(
                                          -USER => ‘anonymous’,
                                          -HOST => ‘mysql-eg-publicsql.ebi.ac.uk’,
                                          -PORT => ‘4157’,
                                         );
2. Set species and chromosome of interest and print BED file with repeats (recipe A4). Ensembl uses 1-based inclusive coordinates internally:
my $species = ‘arabidopsis_thaliana’;
                                          my $chrname = ‘chr4’;
                                          my $slice_adaptor =
                                           Bio::EnsEMBL::Registry->
                                           get_adaptor ($species, ‘core’, ‘Slice’);
                                          my $slice = $slice_adaptor->
                                           fetch_by_region( ‘toplevel’, $chrname );
                                          my @repeats = @{ $slice->get_all_RepeatFeatures() };
                                          foreach my $repeat (@repeats) {
                                           printf(“%s\t%d\t%d\t%s\t%s\t%s\n”,
                                           $chrname,
                                           $repeat->start()-1,
                                           $repeat->end(),
                                           $repeat->analysis()->logic_name(),
                                           $repeat->repeat_consensus()->repeat_class(),
                                           $repeat->repeat_consensus()->repeat_type() );
                                          }

#### 3.2.2 Get Markers Mapped on Chromosome 1D of Bread Wheat

Only a few plants have markers loaded. Recipe A8 retrieves wheat KASP markers, with coordinates returned in BED format:
$species = ‘triticum_aestivum’;
                                          $chrname = ‘1D’;
                                          $slice_adaptor =
                                           Bio::EnsEMBL::Registry->
                                           get_adaptor( $species, ‘Core’, ‘Slice’ );
                                          $slice = $slice_adaptor->
                                           fetch_by_region( ‘chromosome’, $chrname );
                                          foreach my $mf (@{ $slice->get_all_MarkerFeatures() }) {
                                           my $marker = $mf->marker();
                                           printf(“%s\t%d\t%d\t%s\t%s\t%s\t%d\n”,
                                           $mf->seq_region_name(),
                                           $mf->start()-1,
                                           $mf->end(),
                                           $mf->display_id(),
                                           $marker->left_primer(),
                                           $marker->right_primer(),
                                           $marker->max_primer_dist() );
                                          }

### 3.3 R Biomart Recipes

The BioMart databases can be queried in many ways (*see* Note 6). There are five recipes in the repository written in the R language. They all use the BioConductor package BiomaRt [46], which can be installed as follows:
if (!requireNamespace(“BiocManager”, quietly = TRUE))
                                           install.packages(“BiocManager”)
                                          BiocManager::install(“biomaRt”)

This example corresponds to recipe R4, which queries sunflower genes to obtain annotated Pfam domains. Dataset names are abbreviations of Ensembl production names. See recipe R5 for an example querying BioMart variation databases:
EPgenes = useMart(
                                           biomart=“plants_mart”,
                                           host=“plants.ensembl.org”,
                                           dataset=“hannuus_eg_gene”)
                                          pfam = getBM(
                                           attributes=c(“ensembl_gene_id”, “pfam”),
                                           mart=EPgenes)

### 3.4 FTP Recipes

There are 12 recipes in the repository that query the Ensembl Genomes FTP server. They use shell variables and the *wget* program to download files. The recipes refer to the Ensembl release and the Ensembl Plants release as RELEASE and EGRELEASE, respectively. Recipe F5 involves a prewritten BioMart query.

#### 3.4.1 Download Soft-Masked Genomic Sequences

Soft-masked sequences are FASTA files with all annotated repeated elements in lower case. Using recipe F4 they can be downloaded for a chosen species and release as follows:
SERVER=ftp://ftp.ensemblgenomes.org/pub
                                          DIV=plants
                                          EGRELEASE=47
                                          SPECIES=Brachypodium_distachyon
                                          FASTASM=“${SPECIES}*.dna_sm.toplevel.fa.gz”
                                          URL=“${SERVER}/release-${EGRELEASE}/${DIV}/fasta/${SPE-
                                          CIES,,}/dna/${FASTASM}”
                                          wget -c “$URL”

#### 3.4.2 Download All Homologies in a Single TSV File

Recipe F9 downloads a large file (several GB) with all homologies of a release in TSV format. Sequence identifiers correspond to canonical transcripts (*see* Note 4):
TSVFILE=“Compara.${RELEASE}.protein_default.homologies.tsv.gz”
                                          URL=“${SERVER}/${DIV}/release-${EGRELEASE}/tsv/ensembl-com-para/homologies/${TSVFILE}”
                                          wget -c “$URL”

This file can be parsed in the command line in order to extract homologies (*see* Note 7):
zcat “$TSVFILE” | grep triticum_aestivum | greporyza_sativa |
                                          grep ortholog

Homologies of each species can be retrieved from a smaller, specific file:
TSVFILE=“Compara.${RELEASE}.protein_default.homologies.tsv.gz”
                                          SPECIES=Triticum_aestivum
                                          URL=“${SERVER}/${DIV}/release-${EGRELEASE}/tsv/ensembl-com-
                                          para/homologies/${SPECIES,,}${TSVFILE}”
                                          wget -c “$URL”
                                          zcat “$TSVFILE” | grep oryza_sativa | grep ortholog

Homologies can also be downloaded in OrthoXML format [47], which renders a smaller file but requires a more complex parser.

### 3.5 MySQL Recipes

Direct access to the public MySQL server requires knowledge of the schemas (*see* Notes 1 and 8). While this approach supports complex queries with high-performance, the schemas may change in a new release and thus some queries might stop working. For this reason, API access is recommended. Three recipes are shown here, they all require the *mysql*-client to be installed.

#### 3.5.1 Count Protein-Coding Genes of a Particular Species

This is recipe S2. The source code works out the current release number, but it can also be set manually as in this example:
SERVER=mysql-eg-publicsql.ebi.ac.uk
                                          USER=anonymous
                                          PORT=4157
                                          EGRELEASE=47
                                          RELEASE=$((EGRELEASE + 53))
                                          SPECIES=arabidopsis_thaliana
                                          SPECIESCORE=$(mysql --host $SERVER --user $USER --port $PORT \
                                          -e “show databases” | grep \
                                          “${SPECIES}_core_${EGRELEASE}_${RELEASE}”)
                                         mysql --host $SERVER --user $USER --port $PORT \
                                          $SPECIESCORE -e “SELECT COUNT(*) FROM gene \
                                          WHERE biotype=‘protein_coding’”

#### 3.5.2 Get stable_ids of Transcripts Used in Compara Analyses

Recipe S3 gets a list of identifiers of all transcript used in the comparative genomics gene tree analysis (*see* Note 4):
SERVER=mysql-eg-publicsql.ebi.ac.uk
                                          USER=anonymous
                                          PORT=4157
                                          EGRELEASE=47
                                          RELEASE=$((EGRELEASE + 53))
                                          SPECIES=arabidopsis_thaliana
                                          mysql --host $SERVER --user $USER --port $PORT \
                                           “ensembl_compara_plants_${EGRELEASE}_${RELEASE}” \
                                           -e “SELECT sm.stable_id \
                                           FROM seq_member sm, gene_member gm, genome_db gdb \
                                           WHERE sm.seq_member_id = gm.canonical_member_id \
                                           AND sm.genome_db_id = gdb.genome_db_id \
                                           AND gdb.name = ‘$SPECIES’”

See recipe F3 to obtain the corresponding sequences.

#### 3.5.3 Get Variants Significantly Associated with Phenotypes

Recipe S4 queries several tables of the variation schema (see Note 8):
SPECIESVAR=$(mysql --host $SERVER --user $USER --port $PORT \
                                          -e “show databases” | \
                                          grep “${SPECIES}_variation_${EGRELEASE}_${RELEASE}”)
                                          mysql --host $SERVER --user $USER --port $PORT \
                                           $SPECIESVAR<<SQL
                                           SELECT f.object_id, s.name, f.seq_region_start,
                                           f.seq_region_end, p.description
                                           FROM phenotype p
                                           JOIN phenotype_feature f ON p.phenotype_id = f.phenotype_id
                                           JOIN seq_region s ON f.seq_region_id = s.name
                                           WHERE f.type = ‘Variation’ AND f.is_significant=1
                                          SQL

### 3.6 REST Recipes

The following recipes, written in Python, can also be found in R and Perl languages in the repository. They communicate with the Ensembl REST service at https://rest.ensembl.org (*see* Note 9) using the functions get_json and get_json_post, defined in file *exampleREST.py*.

#### 3.6.1 Find Features Overlapping a Genomic Region

Recipe R3 queries the endpoint overlap/region and returns all features overlapping a selected genomic region:
def get_overlapping_features(species,region):
                                           overlap_url = (“/overlap/region/” + species + “/” + region)
                                           # repeat or variation could have been used instead of gene
                                           ext = (overlap_url + “?feature=gene;content-type=application/
                                          json”)
                                           overlap_data = get_json(ext)
                                           for overlap_feat in overlap_data:
                                           print(“%s\t%s\t%s” % (overlap_feat[‘id’],
                                           overlap_feat[‘start’],
                                           overlap_feat[‘end’]))
                                          species = ‘triticum_aestivum’;
                                          region = ‘3D:379400000-379540000’;
                                          get_overlapping_features(species,region)

#### 3.6.2 Check Consequences of SNPs Within CDS Sequences

Recipe R8 queries two endpoints (map/cds/ and info/vep/:species/region). The first one translates CDS to genomic coordinates, the second one retrieves the predicted consequences of the SNP in the coding sequence. This recipe can be used to annotate genomic variants in a given gene across germplasm panels, as done in [48]:
def check_snp_consequences(species,transcript_id,SNPCDScoord,
                                          SNPbase):
                                           # convert CDS coords to genomic coords
                                           ext = (“/map/cds/” + transcript_id + “/”
                                           + SNPCDScoord + “..” + SNPCDScoord
                                           + “?content-type=application/json;species=“ + species)
                                           map_cds = get_json(ext)
                                           if map_cds[‘mappings’][0][‘seq_region_name’]:
                                           mapping = map_cds[‘mappings’][0]
                                           # fetch VEP consequences for this region
                                           SNPgenome_coord = ( mapping[‘seq_region_name’] + ‘:’ +
                                           str(mapping[‘start’]) + ‘-’ + str(mapping[‘end’]) )
                                           ext = (“/vep/”+ species + “/region/” + SNPgenome_coord + “/” +
                                           SNPbase + “?content-type=application/json”)
                                           conseq = get_json(ext)
                                           # Print all the relevant info for the given variant
                                           if conseq[0][‘allele_string’]:
                                           for tcons in conseq[0][‘transcript_consequences’]:
                                           #... some lines omitted, check exampleREST.py
                                           values = (transcript_id, SNPCDScoord,
                                           conseq[0][‘allele_string’],
                                           tcons[‘biotype’],
                                           tcons[‘codons’],
                                           tcons[‘amino_acids’],
                                           tcons[‘protein_start’],
                                           tcons[‘impact’],
                                           tcons[‘sift_prediction’],
                                           tcons[‘sift_score’])
                                           for val in values:
                                           print (val, end=“\t”)
                                           print()
                                           species = ‘triticum_aestivum’
                                           transcript_id = ‘TraesCS4B02G042700.1’
                                           SNPCDScoord = ‘812’
                                           SNPbase = ‘T’
                                           check_snp_consequences(species,transcript_id,SNPCDScoord,
                                           SNPbase)

### 3.7 Annotate the Effect of Variants with the Ensembl Variant Effect Predictor

The Ensembl VEP tool can be used to predict the effect of variants on genes, transcripts, and protein sequences (*see* Note 10). As mentioned in Table 2, this analysis is run for all genomic variants imported into Ensembl (*see* Table 5). While the Ensembl VEP is available through a web interface, the advantage of a local installation is that it can be used to analyze variation sets of any species, including species that are not in Ensembl Plants. If variants are mapped to a reference genome supported in Ensembl Plants, using a cache file increases performance. However, as shown in recipe V4, it is possible to use other reference FASTA files together with the corresponding GFF/GTF annotation files. The next steps summarize how the software is installed and used following recipes F8, V1, V2, and V3.

1. Clone the repository: git clone https://github.com/Ensembl/ensembl-vep.git.
2. Navigate to the Ensembl VEP directory: cd ensembl-vep.
3. Install Ensembl VEP: perl INSTALL.pl.
4. Download cache file with recipe F8
SPECIES=arabidopsis_thaliana
                                          VEPCACHE=“${SPECIES,,}*.tar.gz*”
                                          URL=“${SERVER}/${DIV}/release-${EGRELEASE}/variation/vep/
                                          ${VEPCACHE}”
                                          wget -c “$URL”
5. Unpack downloaded cache file and check SIFT support:
tar xfz $VEPCACHE
                                          grep sift “${SPECIES}/${EGRELEASE}_*/info.txt”
6. Predict effect of variants, *see* Note 11:
EGRELEASE=47
                                          VCFILE=ensembl-vep/examples/arabidopsis_thaliana.TAIR10.vcf
                                          VEPOPTIONS=(
                                           --genomes # Ensembl Genomes, for Plants
                                           --species $SPECIES
                                           --cache # use local cache file, opposed to --database
                                           --dir_cache ./ # path of unpacked cache $SPECIES folder
                                           --cache_version $EGRELEASE
                                           --input_file $VCFILE
                                           --output_file ${VCFILE}.vep
                                           --check_existing # co-located known variants
                                           --distance 5000 # max dist between variant and transcript
                                           --biotype # show biotype of neighbor transcript
                                          )
                                          ensembl-vep/vep “${VEPOPTIONS[@]”

### 3.8 Querying Plant Pangenomes

**Fig. 2:**
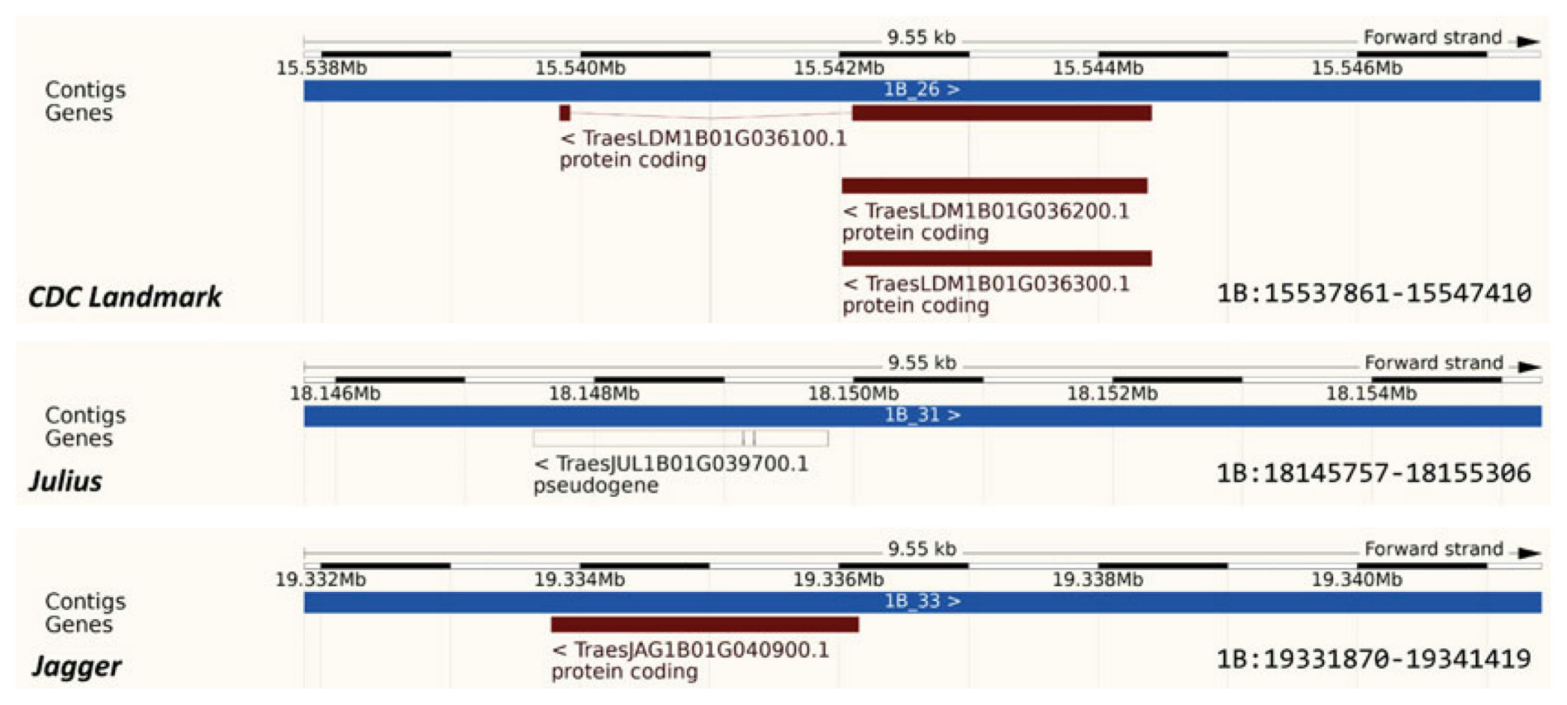
The *RFL* gene (TraesCS1B02G038500) lifted over from the reference landrace Chinese Spring to three wheat cultivars (CDC Landmark, Julius and Jagger). The genes are displayed in the Ensembl Plants genome browser. While in the first cultivar there are three annotated transcript isoforms including one with two exons, the others have a single transcript with one exon. Furthermore, the locus is annotated as a pseudogene in Julius

Upcoming Ensembl Plants releases will have an increasing number of species with multiple cultivars or ecotypes as additional assemblies are added in collaboration with the relevant communities. On the website these cultivars can be browsed from the appropriate reference genome page such as http://plants.ensembl.org/Triticum_aestivum/Info/Strains?db=core (*see* Note 12). Starting with several UK cultivars in release 48 (August 2020), Ensembl will host all cultivars of the first assembled wheat pangenome [49] from release 50 planned for early 2021 (*see* example Fig. 2). Note that related noncultivated species are often included in the pangenomes of crops. For example, Ensembl Plants hosts 11 *Oryza* species plus the outgroup plant *Leersia perrieri*. Both types of genome sets can be considered pangenomes.

Currently, some pangenomes in Ensembl can be interrogated using gene trees and whole-genome alignments (WGAs; *see* Tables 2 and 6). For example, recipe A9 can be used to retrieve syntenic orthologous genes in rice or *Brassicaceae* species. These analyses will be available for wheat as well once de novo gene annotation and WGAs are produced.

### 3.9 Getting Help

Documentation for Ensembl Plants, including FAQs, tutorials, and detailed information about the project, datasets, and pipelines that we run can be found under the “Documentation” and “Website help” links at the top of every page. Detailed information for each species can be found on the species homepage. The EMBL-EBI train online website has several free courses on Ensembl, including the recently updated “Ensembl Genomes (non-chordates): Quick tour” (https://www.ebi.ac.uk/training/online/course/ensembl-genomes-non-chordates-quick-tour) and “Ensembl REST API” courses (https://www.ebi.ac.uk/training-beta/online/courses/ensembl-rest-api). Any data problems are reported on our blog http://www.ensembl.info/known-bugs. If the available documentation cannot answer your question, a helpdesk is provided (mail helpdesk@ensemblgenomes.org with your query).

## Figures and Tables

**Table 1:** Programming interfaces and data sources in Ensembl Plants. The public MySQL server contains databases from the most recent ten releases

Resource	Description
Perl API	A comprehensive Perl-based API for accessing all types of data available: http://plants.ensembl.org/info/docs/api/index.html
REST service	A language-independent API for retrieving selected data: http://plants.ensembl.org/info/data/rest.html
BioMart	A data mining tool for batch retrieval of gene-related data. Accessible via web interface and a Bioconductor package: http://plants.ensembl.org/info/data/biomart/index.html
FTP server	Pre-generated genome-scale data files in a variety of commonly used formats: http://plants.ensembl.org/info/data/ftp/index.html
MySQL server	Public access to Ensembl Genomes MySQL databases: http://plants.ensembl.org/info/data/mysql.html

**Table 2:** Standard computational analyses that are typically run for genomes in Ensembl Plants. The full list of analyses for any species can be obtained with recipe A2

Analysis	Description
Repeat classification and masking	Several tools for detecting and classifying repeated elements are used: http://plants.ensembl.org/info/genome/annotation/repeat_features.html
RNA gene	Noncoding genes are primarily annotated by homology-based methods: http://plants.ensembl.org/info/genome/annotation/ncrna.html
External cross-references	Database cross-references are loaded from a predefined set of sources, using either direct mappings or sequence alignments [7, 14]: http://plants.ensembl.org/info/genome/annotation/cross_references.html
Ontology terms	Ontology terms are imported from external sources and also transitively annotated via InterPro [7]: http://plants.ensembl.org/info/genome/annotation/cross_references.html
Plant Reactome	Metabolic, transport, and hormone signaling pathways, transcriptional networks, and developmental processes [15]: https://plantreactome.gramene.org
Protein features	InterProScan provides protein domain and feature annotations: http://plants.ensembl.org/info/genome/annotation/protein_features.html
Gene trees	Comparative genomics pipeline that computes phylogenetic trees of protein-coding genes [16]: http://plants.ensembl.org/info/genome/compara/peptide_compara.html
Whole-genome alignment (WGA)	Whole-genome alignments are computed for selected pairs of species. When both genomes permit, synteny calculations are also performed. *See* http://plants.ensembl.org/info/genome/compara/whole_genome_alignment.html and http://plants.ensembl.org/info/genome/compara/synteny.html
Variation coding consequences	The consequences of polymorphisms in species with variation datasets are computed for each transcript with the Ensembl Variant Effect Predictor [14]: http://plants.ensembl.org/info/docs/tools/vep

**Table 3:** Genomes available in release 47 (April 2020) of Ensembl Plants. The chr column indicates chromosome-level assemblies. The base count of the genome golden path is given in Mbp. This table was produced with recipe R2

Ensembl production name	Cultivar/ecotype	Assembly	chr	Base count
actinidia_chinensis	Red5	GCA_003024255.1	Y	553.8
aegilops_tauschii	AL8/78	GCA_002575655.1	Y	4224.9
amborella_trichopoda	NA	GCA_000471905.1		706.3
ananas_comosus	F153	GCA_902162155.1		315.8
arabidopsis_halleri	W302	GCA_900078215.1		196.2
arabidopsis_lyrata	MN47	GCA_000004255.1	Y	206.7
arabidopsis_thaliana	Columbia	GCA_000001735.1	Y	119.7
beta_vulgaris	KWS2320 DH	GCA_000511025.2	Y	566.2
brachypodium_distachyon	Bd21	GCA_000005505.4	Y	271.2
brassica_napus	Darmor-bzh	GCA_000751015.1		848.2
brassica_oleracea	TO1000	GCA_000695525.1	Y	488.6
brassica_rapa	Chiifu-401-42	GCA_000309985.1	Y	283.8
capsicum_annuum	Criollo de Morelos 334	GCA_000512255.2	Y	3063.9
chara_braunii	S276	GCA_003427395.1		1751.2
chlamydomonas_reinhardtii	CC-503 cw92 mt+	GCA_000002595.3	Y	111.1
chondrus_crispus	Stackhouse	GCA_000350225.2	Y	105
citrus_clementina	Clemenules	GCA_000493195.1		301.4
coffea_canephora	DH200-94	GCA_900059795.1	Y	568.6
corchorus_capsularis	CVL-1	GCA_001974805.1		317.2
cucumis_sativus	9930	GCA_000004075.2	Y	193.8
cyanidioschyzon_merolae	10D	GCA_000091205.1	Y	16.7
cynara_cardunculus	NA	GCA_001531365.1		724.7
daucus_carota	DH1	GCA_001625215.1	Y	421.5
dioscorea_rotundata	TDr96_F1	GCA_002240015.2	Y	456.7
eragrostis_curvula	Tanganyika	GCA_007726485.1	Y	603.1
eragrostis_tef	Tsedey	GCA_000970635.1		607.3
galdieria_sulphuraria	074W	GCA_000341285.1		13.7
glycine_max	Williams 82	GCA_000004515.4	Y	978.5
gossypium_raimondii	CMD 10	GCA_000327365.1	Y	761.4
helianthus_annuus	XRQ/B	GCA_002127325.1	Y	3027.8
hordeum_vulgare	Morex	GCA_901482405.1	Y	4834.4
ipomoea_triloba	NCNSP0323	GCA_003576645.1	Y	461.8
leersia_perrieri	IRGC:105164	GCA_000325765.3	Y	266.7
lupinus_angustifolius	Tanjil	GCA_001865875.1	Y	609.2
malus_domestica	Golden Delicious	GCA_002114115.1	Y	703
manihot_esculenta	AM560-2	GCA_001659605.1	Y	582.1
marchantia_polymorpha	Tak-1	GCA_003032435.1		225.8
medicago_truncatula	A17	GCA_000219495.2	Y	412.8
musa_acuminata	DH-Pahang	GCA_000313855.1	Y	473
nicotiana_attenuata	UT	GCA_001879085.1	Y	2365.7
olea_europaea_sylvestris	NA	GCA_002742605.1	Y	1141
oryza_barthii	IRGC:105608	GCA_000182155.2	Y	308.3
oryza_brachyantha	IRGC:101232	GCA_000231095.2	Y	260.8
oryza_glaberrima	CG14	GCA_000147395.1	Y	316.4
oryza_glumipatula	NA	GCA_000576495.1	Y	372.9
oryza_indica	93-11	GCA_000004655.2	Y	427
oryza_longistaminata	NA	GCA_000789195.1		326.4
oryza_meridionalis	OR44 (W2112)	GCA_000338895.2	Y	335.7
oryza_nivara	IRGC:100897	GCA_000576065.1	Y	338
oryza_punctata	IRGC:105690	GCA_000573905.1	Y	393.8
oryza_rufipogon	W1943	GCA_000817225.1	Y	338
oryza_sativa	Nipponbare	GCA_001433935.1	Y	375
ostreococcus_lucimarinus	CCE9901	GCA_000092065.1	Y	13.2
panicum_hallii_fil2	FIL2	GCA_002211085.2	Y	535.9
panicum_hallii_hal2	HAL2	GCA_003061485.1	Y	487.5
phaseolus_vulgaris	G19833	GCA_000499845.1	Y	521.1
physcomitrella_patens	Gransden 2004	GCA_000002425.2	Y	471.9
pistacia_vera	Batoury	GCA_008641045.1		671.2
populus_trichocarpa	Nisqually 1	GCA_000002775.3	Y	434.1
prunus_avium	Satonishiki	GCA_002207925.1		272.4
prunus_dulcis	Texas	GCA_902201215.1		227.5
prunus_persica	Lovell	GCA_000346465.2	Y	227.4
saccharum_spontaneum	AP85-441	GCA_003544955.1	Y	2900.2
selaginella_moellendorffii	NA	GCA_000143415.1		212.6
setaria_italica	Yugu1	GCA_000263155.2	Y	405.7
solanum_lycopersicum	Heinz 1706	GCA_000188115.3	Y	827.7
solanum_tuberosum	DM 1-3 516 R44	GCA_000226075.1	Y	810.7
sorghum_bicolor	BTx623	GCA_000003195.3	Y	708.7
theobroma_cacao_criollo	Criollo B97-61/B2	GCA_000208745.2	Y	324.7
theobroma_cacao_matina	Matina 1-6	GCA_000403535.1	Y	346
trifolium_pratense	Milvus B	GCA_900079335.1	Y	304.8
triticum_aestivum	Chinese spring	GCA_900519105.1	Y	14547.3
triticum_dicoccoides	Zavitan (Atlit2015)	GCA_002162155.1	Y	10079
triticum_turgidum	svevo	GCA_900231445.1	Y	10463.1
triticum_urartu	G1812 (PI428198)	GCA_000347455.1	Y	3747.2
vigna_angularis	Jingnong 6	GCA_001190045.1	Y	466.7
vigna_radiata	VC1973A	GCA_000741045.2	Y	463.1
vitis_vinifera	PN40024	GCA_000003745.2	Y	486.3
zea_mays	B73	GCA_000005005.6	Y	2135.1

**Table 4:** Protein-coding genes annotated in release 47 (April 2020) of Ensembl Plants. The last two columns indicate how many genes encode proteins computationally predicted (TrEMBL) and manually curated (SwissProt) in UniProtKB. This table was produced with recipe F10. Mappings between Ensembl and UniProt proteins can be obtained with recipe F6

Ensembl production name	Protein-coding genes	TrEMBL	SwissProt
actinidia_chinensis	33,044	33,044	6
aegilops_tauschii	39,614	24,486	7
amborella_trichopoda	27,313	27,310	34
ananas_comosus	25,783	16,219	8
arabidopsis_halleri	32,158	241	0
arabidopsis_lyrata	32,667	32,470	30
arabidopsis_thaliana	27,628	27,100	15,649
beta_vulgaris	26,521	7,405	37
brachypodium_distachyon	34,310	34,307	36
brassica_napus	101,040	62,919	149
brassica_oleracea	59,220	59,220	20
brassica_rapa	41,018	141	9
capsicum_annuum	35,845	35,845	52
chara_braunii	34,718	33,777	0
chlamydomonas_reinhardtii	17,743	17,737	322
chondrus_crispus	9,807	9,806	11
citrus_clementina	25,000	24,989	0
coffea_canephora	25,574	25,574	3
corchorus_capsularis	29,356	29,356	0
cucumis_sativus	23,780	23,780	65
cyanidioschyzon_merolae	4,973	4,640	97
cynara_cardunculus	26,505	26,504	6
daucus_carota	32,109	32,109	136
dioscorea_rotundata	19,023	13	0
eragrostis_curvula	55,182	2	0
eragrostis_tef	41,555	54	0
galdieria_sulphuraria	6,622	6,621	23
glycine_max	55,897	55,891	412
gossypium_raimondii	38,208	38,172	0
helianthus_annuus	52,191	52,191	315
hordeum_vulgare	37,705	37,636	292
ipomoea_triloba	31,358	0	0
leersia_perrieri	29,078	29,074	0
lupinus_angustifolius	33,074	14,421	12
malus_domestica	40,624	28,704	41
manihot_esculenta	33,044	33,043	45
marchantia_polymorpha	19,287	19,287	76
medicago_truncatula	50,444	50,431	79
musa_acuminata	36,519	36,519	11
nicotiana_attenuata	33,320	33,320	3
olea_europaea_sylvestris	50,678	333	23
oryza_barthii	34,575	34,564	0
oryza_brachyantha	32,037	32,032	0
oryza_glaberrima	33,164	33,161	1
oryza_glumipatula	35,735	35,721	0
oryza_indica	40,745	36,796	570
oryza_longistaminata	31,686	101	0
oryza_meridionalis	29,308	29,294	0
oryza_nivara	36,313	36,305	27
oryza_punctata	31,762	31,748	0
oryza_rufipogon	37,071	37,063	1
oryza_sativa	35,775	32,864	3,096
ostreococcus_lucimarinus	7,603	7,570	20
panicum_hallii_fil2	33,805	33,805	0
panicum_hallii_hal2	33,263	33,263	0
phaseolus_vulgaris	28,134	28,095	111
physcomitrella_patens	32,234	0	0
pistacia_vera	31,784	43	0
populus_trichocarpa	41,335	41,335	135
prunus_avium	42,794	219	8
prunus_dulcis	27,963	27,963	10
prunus_persica	26,873	26,873	17
saccharum_spontaneum	53,284	65	0
selaginella_moellendorffii	34,799	34,762	31
setaria_italica	35,831	35,828	2
solanum_lycopersicum	34,429	27,133	406
solanum_tuberosum	39,021	39,010	245
sorghum_bicolor	34,118	34,078	142
theobroma_cacao_criollo	21,146	4,079	5
theobroma_cacao_matina	29,188	29,188	5
trifolium_pratense	39,917	26,935	0
triticum_aestivum	107,545	107,124	600
triticum_dicoccoides	62,569	182	1
triticum_turgidum	66,545	233	0
triticum_urartu	33,482	33,479	1
vigna_angularis	33,860	33,860	1
vigna_radiata	22,368	5,978	0
vitis_vinifera	29,927	29,814	136
zea_mays	39,591	39,494	724

**Table 5:** Variation datasets available in release 47 (April 2020) of Ensembl Plants. The list can also be browsed interactively at https://plants.ensembl.org/species.html. This table was produced with recipe R9. The corresponding VCF files can be downloaded with recipe F7. Recipe F8 can be used to get the Ensembl VEP cache files in order to annotate variant consequences with recipes V2 and V3

Ensembl production name	Source
arabidopsis_thaliana	The 1001 Genomes Project [18]
arabidopsis_thaliana	Nordborg [19]
brachypodium_distachyon	Jaiswal_lab_OSU [20]
hordeum_vulgare	International Barley Sequencing Consortium (IBSC) [21–23]
hordeum_vulgare	Ensembl Plants [24]
hordeum_vulgare	IlluminaiSelect SNP chip [22]
malus_domestica	http://fruitbreedomics.com [25]
oryza_glaberrima	Glab (OGE)
oryza_glaberrima	Barthii(OGE)
oryza_glumipatula	Oryza Genome Evolution (OGE)
oryza_indica	dbSNP [26]
oryza_sativa	https://www.ebi.ac.uk/eva [27–30]
oryza_sativa	https://archive.gramene.org/qtl (Gramene_QTLdb) [6]
oryza_sativa	https://archive.gramene.org/markers (gramene-marker) [6]
oryza_sativa	Qtaro_QTLdb [31]
solanum_lycopersicum	The 150 Tomato Genome ReSequencing Project [32]
sorghum_bicolor	Morris_2013 [33]
sorghum_bicolor	Database of Genomic Variants Archive (DGVa)
sorghum_bicolor	Mace_2013 [34]
sorghum_bicolor	Sorghum_EMS_mutants [35]
triticum_aestivum	Markers from Axiom 820K and 35K SNP Array provided (CerealsDB) [36]
triticum_aestivum	EMS-induced mutation [37]
triticum_aestivum	Inter-Homoeologous Variants (IHVs) called by alignments ofthe A, B, and D component genomes
triticum_turgidum	Markers from Axiom 820K, 35K, iSelect 90KSNP Infinium and TaBW280K Affymetrix array (CNR-ITB) [36, 38]
vitis_vinifera	CSHL/Cornell [39]
zea_mays	HapMap2 [40]
zea_mays	Panzea_2.7GBS https://www.panzea.org/genotypes

**Table 6:** Number of pairwise whole-genome alignments and synteny analyses in release 47 (April 2020) of Ensembl Plants. Pairwise alignments are computed with LastZ [43]. Two multiple alignments are also available for *Oryza* species. Data obtained with recipe S6

Ensembl production name	WGA pairwise alignments	Synteny analyses
actinidia_chinensis	4	2
aegilops_tauschii	8	3
amborella_trichopoda	3	0
ananas_comosus	3	0
arabidopsis_halleri	4	0
arabidopsis_lyrata	5	2
arabidopsis_thaliana	77	4
beta_vulgaris	3	0
brachypodium_distachyon	10	1
brassica_napus	5	0
brassica_oleracea	6	0
brassica_rapa	6	1
capsicum_annuum	4	1
chara_braunii	0	0
chlamydomonas_reinhardtii	3	0
chondrus_crispus	3	0
citrus_clementina	4	0
coffea_canephora	4	2
corchorus_capsularis	5	0
cucumis_sativus	4	0
cyanidioschyzon_merolae	3	0
cynara_cardunculus	4	0
daucus_carota	4	0
dioscorea_rotundata	3	0
eragrostis_curvula	3	1
eragrostis_tef	3	0
galdieria_sulphuraria	3	0
glycine_max	4	0
gossypium_raimondii	5	0
helianthus_annuus	4	0
hordeum_vulgare	9	0
ipomoea_triloba	4	0
leersia_perrieri	11	2
lupinus_angustifolius	4	0
malus_domestica	4	0
manihot_esculenta	4	0
marchantia_polymorpha	3	0
medicago_truncatula	22	4
musa_acuminata	5	1
nicotiana_attenuata	4	0
olea_europaea_sylvestris	4	0
oryza_barthii	13	9
oryza_brachyantha	12	9
oryza_glaberrima	13	9
oryza_glumipatula	13	9
oryza_indica	13	9
oryza_longistaminata	13	0
oryza_meridionalis	13	10
oryza_nivara	13	9
oryza_punctata	12	9
oryza_rufipogon	13	9
oryza_sativa	77	20
ostreococcus_lucimarinus	3	0
panicum_hallii_fil2	3	1
panicum_hallii_hal2	3	1
phaseolus_vulgaris	4	1
physcomitrella_patens	4	0
pistacia_vera	4	0
populus_trichocarpa	4	0
prunus_avium	4	0
prunus_dulcis	4	0
prunus_persica	4	2
saccharum_spontaneum	3	0
selaginella_moellendorffii	3	0
setaria_italica	4	1
solanum_lycopersicum	13	4
solanum_tuberosum	4	2
sorghum_bicolor	6	1
theobroma_cacao_criollo	13	2
theobroma_cacao_matina	4	2
trifolium_pratense	4	0
triticum_aestivum	9	3
triticum_dicoccoides	9	3
triticum_turgidum	8	3
triticum_urartu	4	0
vigna_angularis	5	2
vigna_radiata	5	2
vitis_vinifera	77	9
zea_mays	8	1

**Table 7:** Programming recipes to analyze data in Ensembl Plants, including perl API (A), R BiomaRt (B), FTP (F), SQL (S), REST (R), and Ensembl VEP (V) examples. These recipes and their software dependencies, together with a few more scripts for phylogenomic analyses, are updated at https://github.com/Ensembl/plant-scripts

Recipe	Description
A1	Load the Registry object with details of genomes available
A2	Check which analyses are available for a species
A3	Get soft-masked sequences from Arabidopsis thaliana
A4	Get BED file with repeats in chr4
A5	Find the DEAR3 gene
A6	Get the transcript used in Compara analyses
A7	Find all orthologues of a gene
A8	Get markers mapped on chr1D of bread wheat
A9	Find all syntelogues among rices
A10	Print all translations for other features genes
B1	Check plant marts and select dataset
B2	Check available filters and attributes
B3	Download GO terms associated with genes
B4	Get Pfam domains annotated in genes
B5	Get SNP consequences from a selected variation source
C1	Find RNA-seq CRAM files for a genome assembly
F1	Download peptide sequences in FASTA format
F2	Download CDS nucleotide sequences in FASTA format
F3	Download transcripts (cDNA)
F4	Download soft-masked genomic sequences
F5	Upstream/downstream sequences
F6	Get mappings to UniProt proteins
F7	Get indexed, bgzipped VCF file with variants mapped
F8	Get precomputed VEP cache files
F9	Download all homologies in a single TSV file, several GBs
F10	Download UniProt report of Ensembl Plants
F11	Retrieve list of new species in current release
F12	Get current plant species tree cladogram
S1	Check currently supported Ensembl Genomes (EG) core schemas
S2	Count protein-coding genes of a particular species
S3	Get stable_ids of transcripts used in Compara analyses
S4	Get variants significantly associated to phenotypes
S5	Get Triticumaestivumhomeologous genes across A, B, and D subgenomes
S6	Count the number of whole-genome alignments of all genomes
S7	Extract all the mutations and consequence for a known line on triticum_aestivum
R1	Create an HTTP client and helper functions
R2	Get metadata for all plant species
R3	Find features overlapping genomic region
R4	Fetch phenotypes overlapping genomic region
R5	Find homologues of selected gene
R6	Get annotation of orthologous genes/proteins
R7	Fetch variant consequences for multiple variant ids
R8	Check consequences of single SNP within CDS sequence
R9	Retrieve variation sources of a species
V1	Download, install, and update VEP
V2	Unpack downloaded cache file and check SIFT support
V3	Predict effect of variants
V4	Predict effect of variants for species not in Ensembl

## Footnotes

1 The core schema is fully described at https://www.ensembl.org/info/docs/api/core/core_schema.html. There are similar documents for variation, comparative genomics, and regulation schemas: https://www.ensembl.org/info/docs/api/variation/variation_schema.html
https://www.ensembl.org/info/docs/api/compara/compara_schema.html

https://www.ensembl.org/info/docs/api/funcgen/funcgen_schema.html
The schema for the metadata database can be found at https://github.com/Ensembl/ensembl-metadata.

2 Instructions to set up a local Ensembl database are provided at http://plants.ensembl.org/info/docs/webcode/mirror/install/ensembl-data.html.

3 Check the annotation page for each species in Ensembl Plants. For *Arabidopsis thaliana*, this is http://plants.ensembl.org/Arabidopsis_thaliana/Info/Annotation/#genebuild.

4 Gene trees use canonical transcripts, defined at http://plants.ensembl.org/info/website/glossary.html. In plant species, the canonical transcript of a protein-coding gene is the transcript with the longest translation with no stop codons. This does not necessarily reflect the most biologically relevant transcript of a gene. The script https://github.com/Ensembl/plant_tools/blob/master/phylogenomics/ens_sequences.pl can be used to obtain sequences of canonical transcripts in FASTA format.

5 Check http://plants.ensembl.org/info/docs/Doxygen. See also debugging instructions and tutorials at http://plants.ensembl.org/info/docs/api.

6 *See* http://plants.ensembl.org/info/data/biomart.

7 *See* http://plants.ensembl.org/info/genome/compara/homology_method.html for the definitions of the different homology types.

8 The variation schema is described at http://plants.ensembl.org/info/docs/api/variation/variation_schema.html.

9 Training material to learn more about the Ensembl REST interface can be found at https://www.ebi.ac.uk/training/online/course/ensembl-rest-api and https://mybinder.org/v2/gh/Ensembl/rest-api-jupyter-course/master. The different endpoints are documented at https://rest.ensembl.org/documentation.

10 Ensembl VEP functionality can be extended to utilize additional data or run additional analyses using plugins, *see* https://www.ensembl.org/info/docs/tools/vep/script/vep_plugins.html.

11 The full list of options of Ensembl VEP is described at http://www.ensembl.org/info/docs/tools/vep/script/vep_options.html, there are examples at http://www.ensembl.org/info/docs/tools/vep/script/vep_example.html.

12 Reference genomes have binomial production names when possible, such as oryza_sativa (rice) or triticum_aestivum (bread wheat). Additional cultivars or ecotypes have longer trinomial names such as oryza_sativa_indica or triticum_aestivum_cadenza. Following this convention, theobroma_cacao_-matina and panicum_hallii_hal2 will be renamed to theobroma_cacao and panicum_hallii by release 50.
